# The utility of next-generation sequencing in the evaluation of the posterior polymorphous corneal dystrophy 1 locus

**Published:** 2010-12-18

**Authors:** Isabella N. Lai, Vivek S. Yellore, Sylvia A. Rayner, Nerissa C. D’Silva, Catherine K. Nguyen, Anthony J. Aldave

**Affiliations:** The Jules Stein Eye Institute, David Geffen School of Medicine at the University of California, Los Angeles, Los Angeles, CA

## Abstract

**Purpose:**

To identify the genetic basis of posterior polymorphous corneal dystrophy 1 (PPCD1) using next-generation sequencing (NGS) of the common PPCD1 support interval, in which Sanger sequencing failed to identify a pathogenic mutation.

**Methods:**

Enrichment of the portion of chromosome 20 containing the common PPCD1 interval was performed on DNA extracted from an affected and an unaffected member of a family previously linked to the PPCD1 locus. NGS using the Roche 454 Titanium platform was performed, followed by computational analysis using NextGENe Software.

**Results:**

NGS of the selectively enriched chromosomal 20 region between markers D20S48 and D20S190 produced over 400,000 DNA sequence reads with an average of 350 bases for each of the two DNA samples. Alignment of the DNA sequence reads with the reference sequence from the National Center of Biotechnology Information (NCBI) resulted in over 119 million matched bases per sample. Approximately 68,000 DNA sequence variants were identified in the common PPCD1 support interval in the affected individual, which was approximately twice the number of sequence variants identified in the unaffected individual. In both individuals, approximately 0.5% of the identified variants mapped to the 13 known and 16 predicted genes in the PPCD1 support interval, including 16 of the 17 (94%) variants previously identified by Sanger sequencing in the 13 known genes. In both individuals, the variant not identified by NGS was located in a region of inadequate coverage.

**Conclusions:**

NGS identified all of the exonic sequence variants that were previously identified by Sanger sequencing in known genes in adequately covered regions of the common PPCD1 interval, although the pathogenic variant is yet to be discovered. Given adequate coverage of a selectively enriched chromosomal region of interest, NGS represents a useful technique to screen for sequence variants in candidate gene loci that has multiple advantages over previously employed techniques for mutation discovery.

## Introduction

Since the introduction of DNA sequencing in the mid-seventies by Frederick Sanger [1], the sequencing methodology that bears his name has enabled researchers to make significant discoveries and advances in all branches of biology and medicine, including the sequencing of the human genome [2]. While Sanger sequencing has been considered the gold standard for accuracy in generating sequencing data for over three decades, the development of new sequencing methods that are less expensive, less laborious and generate significantly more information in considerably less time have challenged the supremacy of Sanger sequencing in recent years. These new methods are termed next generation sequencing (NGS), characterized by massive parallel sequencing that produces up to a million reads in one run. NGS has been employed for varied applications including resequencing of the human genome [2,3], whole genome resequencing for evolutionary studies [4,5], de novo sequencing of bacterial genomes [6], quantifying of rare transcripts [7], identifying alternative splicing and sequence variation [8], single nucleotide polymorphism (SNP) discovery [9], and targeted resequencing of specific genetic loci [10-12].

We have recently reported the absence of coding region mutations in the positional candidate genes mapped to the common posterior polymorphous corneal dystrophy 1 (PPCD1; OMIM 122000) interval [13]. Although screening of the exonic and intron/exon boundary regions for each of the known and predicted genes in the common PPCD1 interval using Sanger sequencing did not reveal a pathogenic mutation, convincing evidence indicates that the causative mutation(s) for PPCD1 lie(s) within the 2.4 cM region between markers D20S182 and D20S139 that includes 13 known and 16 predicted genes (build 37.1) [13]. Given the fact that Sanger sequencing may miss low frequency sequence variations [14], and in light of reports of the successful application of NGS to identify causative mutations in candidate regions of interest [11,12,15], we sought to determine the utility of NGS in identifying the genetic basis of PPCD1 following enrichment of the region of chromosome 20 containing the common PPCD1 interval.

## Methods

The researchers followed the tenets of the Declaration of Helsinki in the treatment of the subjects reported herein. Study approval was obtained from the Institutional Review Board at the University of California, Los Angeles (UCLA IRB #02–10–092–11).

### Patient identification/DNA collection and isolation

The diagnosis of PPCD was based on established, previously published criteria [16]. A peripheral blood sample was collected from an affected and an unaffected member of a family previously linked to the PPCD1 locus, and DNA was isolated using a commercially-available kit (Flexigene DNA isolation kit; Qiagen, Valencia, CA).

### Sanger sequencing of newly annotated genes in the common PPCD1 interval

We have previously reported the results of Sanger sequencing of the genes mapped to the common PPCD1 interval (build 36.3) [13]. In the most recent build (build 37.1), four additional genes have been mapped to this interval (*LOC100287054*, cytochrome c oxidase assembly factor-like [*PET117*], *LOC100287095*, and *LOC100270804*) and one gene that appeared in the previous build (36.3) was removed (*LOC100130062*). Therefore, using DNA from the same two affected and unaffected individuals that were the source of DNA in our previous report [13], we performed PCR amplification of the coding regions of the recently annotated genes using custom-designed primers (Primer 3; Table 1), and Sanger sequencing as described previously [13].

**Table 1:** Primers used for sequencing of additional genes mapped to common PPCD1 interval in build 37.1.

**Gene**	**Exon**	**Forward primer**	**Tm (ºC)**	**Reverse primer**	**Tm (ºC)**	**Product size (bp)**
LOC100270804*	1A	gcgtggtaatgtggctttgtacc	68.1	tcaacagtaaacgctgcacatcc	68.1	558
	1B	actcgctccttcccgcaaatgta	71.4	ttcttttcagtcgacacatgcaa	66.6	592
	1C	ctccctgacagacactggcctta	68.6	ggctacaaagagccccttcttga	68.4	552
	1D	aagcggatgacctgtgttcactc	68.6	cggtcctgaggtagggctacagt	68.4	527
	1E	agagggtcctgtcatccattgaa	67.8	ccgaactgtaccaaactcatgtgc	68.4	695
LOC100287054	1	gctgttgctgaccagggtgt	67.7	aggctcttctccctcccttgaat	68.2	299
	2	caaaaggacacagaggtgaactgg	67.8	ccatgaccaaccgatgctgt	68.3	486
	3	cacaacattgttccacggtctca	69.1	gcagacagggcagcctcaag	69.7	398
	4	ctggaggggagagggagagaag	68.6	agtagcgccgagaaatccgttac	68.1	395
LOC100287095	1	gcttgtgcctccagaccagaat	68.7	ccaccttggcctcccaaa	68.4	300
	2	ttaaaattgcccaaaacccaagg	68.0	tcacccacgtgcgatatttcttc	68.9	661
PET117	1	ctatgctcggctctcgattgct	68.7	accgcgggggaaagacac	69.6	384
	2	aactgggtatttggaatctgaaa	62.3	tgatcaagtttaaaaggacagtgacca	67.2	394

*The 2017 bp exon of LOC100270804 was amplified and sequenced as 5 overlapping segments.

### Sequence capture and enrichment of common PPCD1 interval

A 4.8 Mb region between D20S48 and D20S190 that contains the 2.4 cM PPCD1 common interval region was selected for enrichment by the Roche-NimbleGen SeqCap Service Workflow. A custom Sequence Capture 385K Human Array was designed and manufactured by Roche NimbleGen (Madison, WI). A total of 385,000 unique, overlapping probes were designed across the PPCD1 target region (Chromosome 20: 17,000,000– 21,258,707; NCBI build 36.1, hg18). The targeted region was tiled so as to avoid capturing repetitive DNA fragments. Approximately 10 μg of genomic DNA was fragmented by sonication to a size range of 300–500 base pairs. The fragmented DNA was purified (AMPure XP system; Agencourt Bioscience Corporation, Beverly, MA) and analyzed on an Agilent Bioanalyzer 2100 (Agilent Technologies, Santa Clara, CA) according to the manufacturer's instructions. The fragments were then ligated to universal gSel3 and gSel4 adapters (Roche NimbleGen) with T4 DNA Ligase. Small fragments (<100 bp) were removed with the use of AMPure Beads (Agencourt Bioscience Corporation). The resulting library was hybridized to the custom 385K array with the use of the NimbleGen Sequence Capture Hybridization System. Hybridized DNA from the PPCD1 target region was washed and eluted with the use of a NimbleGen Wash and Elution Kit according to the manufacturer's instructions. The eluted sample was amplified by ligation-mediated PCR with the use of primers complementary to the sequence of the adaptors.

### Next-generation sequencing

The selectively enriched samples were then sent to Agencourt Bioscience Corporation for NGS on a 454/FLX Genome Sequencer platform (Roche/454 Life Sciences, Brandford, CT) using the GS FLX Titanium service.

### Sequence assembly and analysis

**Figure 1:**
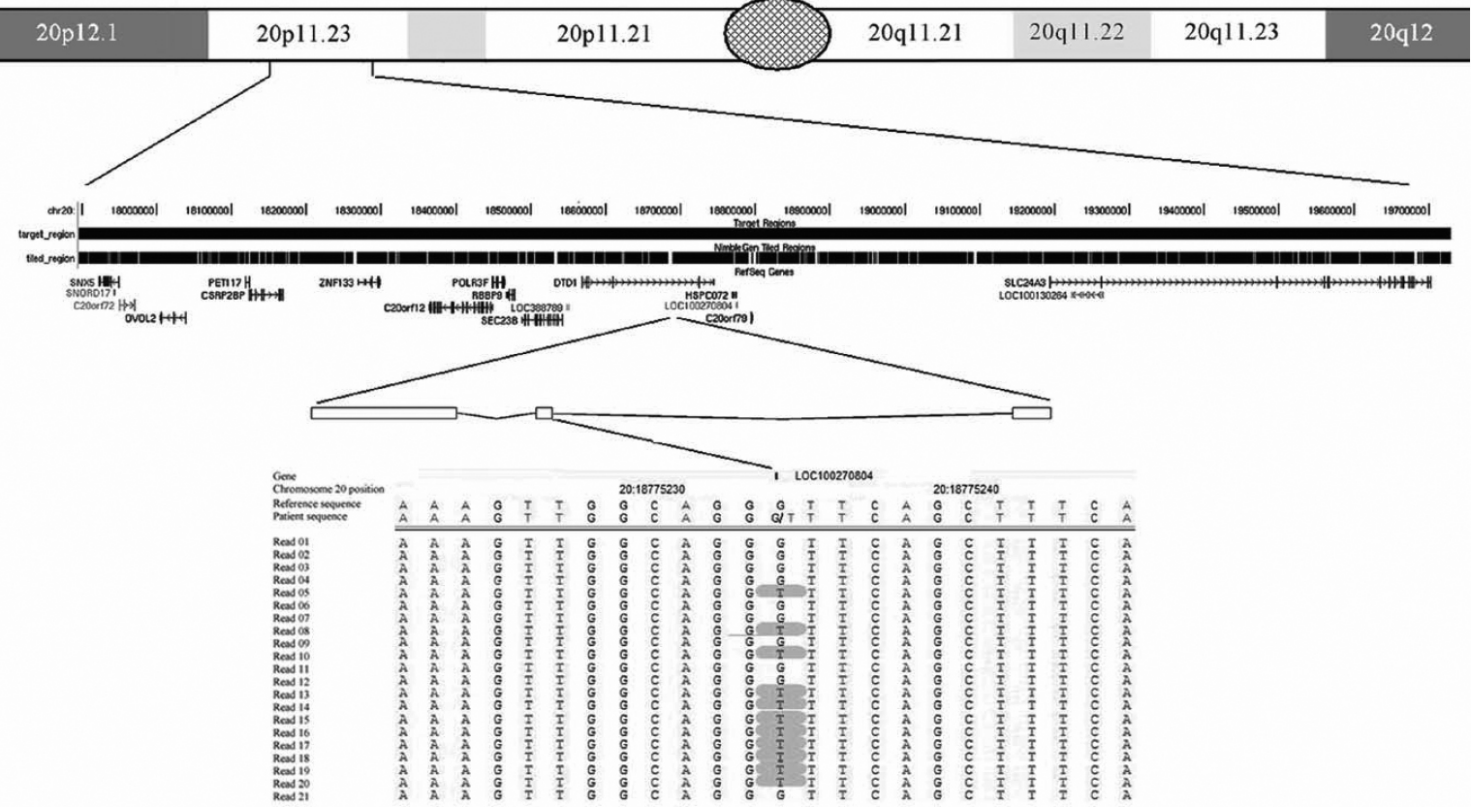
Illustration of pericentromeric region of chromosome 20 to which PPCD1 has been mapped, demonstrating multiple sequence reads that identify a mutation in the gene *LOC100270804*. The common PPCD1 interval is shown within the NimbleGen sequence capture target region as a solid black bar. The bar below it is the tiled region showing breaks where the sequence capture was blocked to prevent binding of repetitive DNA sequences. Also depicted are genes mapped to the common PPCD1 interval, including *LOC100270804*, in which a G>T sequence variant in exon 2 is identified in the heterozygous state.

Sequence data generated by the 454 Genome Sequencer was assembled using NextGENe Software (SoftGenetics, State College, PA) and aligned to the chromosome 20 reference sequence (NCBI build 37.1). Each sequence aligned to a particular genomic region is known as a read, with the number of reads at a certain region being referred to as coverage (Figure 1). The sequence data was analyzed on an Intel i7–920 processor based computer with 12 GB RAM. For the initial analysis that was performed, the default filtering parameters were used with the Condensation Tool (Table 2, Default A), a proprietary software tool that employs depth of coverage to lengthen reads and statistically discount instruments errors (such as homopolymer errors and base call errors caused by pyrosequencing). The data was subsequently analyzed using the default filtering parameters without the Condensation Tool (Table 2, Default B). Data analysis included determining the total number of identified variants, the number of coding region variants in all positional candidate genes, and the number of exonic sequence variants identified in the known genes in the common PPCD1 interval, which were compared to the exonic sequence variants identified in the same genes by Sanger sequencing. The filtering parameters were then adjusted to increase the sensitivity of the software to detect sequence variants in regions with coverage of ≥5 reads (defined as adequate coverage) until all exonic variants identified in the common PPCD1 interval by Sanger sequencing were also identified by NGS (Table 2, Best Adjusted).

**Table 2:** Next-Generation sequencing default parameters run with and without application of Condensation Tool and best adjusted parameters.

**Parameters**	**Default A**	**Default B**	**Best adjusted**
Condensation tool	Yes	No	No
Alignment			
*Seed number*	30	30	35
*Move step*	5	5	10
*Matching base percentage*	80%	80%	85%
Mutation filter (*mutation percentage*)	20%	20%	25%
Mutation score (*optional*)	Not applied	Not applied	Applied, score=5

## Results

### Sanger sequencing of newly annotated genes in the common PPCD1 interval

Sanger sequencing of the newly annotated genes in the common PPCD1 interval (build 37.1) revealed two novel and three previously described SNPs in *LOC100270804* (no variants were identified in *LOC100287054*, *PET117*, or *LOC100287095*). Two of the three known and both of the novel sequence variants in *LOC100270804* were present in both affected family members but not in either unaffected family member that was initially screened. (Figure 1 and Table 3) Screening of nine additional affected family members, nine additional unaffected family members and seven unaffected spouses revealed that the minor allele of each of these four SNPs was present in each of the affected family members and absent in each of the unaffected family members and six of the spouses. The spouse in whom the minor allele of each of these four variants was identified is the same individual in whom the minor allele of three of the four SNPs that we have previously reported as segregating with the affected phenotype in this family were identified [13]. However, none of these SNPs was considered disease-causing as each was identified in unaffected control individuals.

**Table 3:** Sequence variants as identified by Sanger sequencing in candidate genes within the common PPCD1 of Build 37.1 and their appearance in data generated by next-generation sequencing (NGS).

** **	** **	** **	** **	**Next-generation sequencing**		**Sanger sequencing**	
**Gene (refSeq)**	**Nucleotide change**	**Amino acid change**	**refSNP ID**	**Affected**	**Unaffected**	**Affected**	**Unaffected**
SNX5 (NM_152227)	c.543G>A	p.Glu181Glu	rs2273448	Y (hetero) Score=25	Y (hetero)	Y(hetero)	Y (hetero)
**SNORD17 (NR_003045)**	**98T>A**	**Non-coding microRNA**	rs753213	**N**	**Y(hetero) Score=21**	**N**	**Y (hetero)**
C20orf72 (NM_018152)	c.43A>T	p.Ser15Cys	rs11551768	Y (hetero) Score=18	N	Y (hetero)	N
** **	c.794C>T	p.Thr265Ile	-	N	Y (hetero) Score=2**	N	Y (hetero)
** **	c.846T>C	p.Asp282Asp	rs28455091	Y (hetero) Score=2*	Y (hetero) Score=9	Y (hetero)	Y(hetero)
**PTMAP3 (NG_001180)**	**c.347T>G**	**p.Val116Gly**	**-**	N*	N*	**Y (hetero)**	**Y (hetero)**
** **	**c.348C>G**	**p.Val116Val**	**-**	N*	N*	**Y (homo)**	**Y (homo)**
** **	**c.362C>G**	**p.Thr121Arg**	**-**	N*	N*	**Y (homo)**	**Y (homo)**
** **	**c.415T>C**	**p.Leu139Leu**	**-**	N*	N*	**Y (homo)**	**Y (homo)**
** **	**c.553T>C**	**p.Cys185Arg**	rs3748493	N*	N*	**Y (hetero)**	**Y (homo)**
** **	**c.843C>T**	**p.Cys281Cys**	rs3748492	N*	Y (homo) Score=4**	**Y (hetero)**	**Y (homo)**
**LOC100287054**	**No Changes**	** **	** **	** **	** **	** **	** **
OVOL2 (NM_021220)	c.327C>A	p.Thr109Thr	rs6111803	Y (hetero) Score=17	N	Y (hetero)	N
**RPL15P1 (NG_000975)**	**No Changes**	** **	** **	** **	** **	** **	** **
PET117	No Changes	** **	** **	** **	** **	** **	** **
CSRP2BP (NM_020536)	c.1199T>G	p.Val400Gly	rs1205193	Y (homo) Score=23	Y (homo) Score=18	Y (homo)	Y (homo)
** **	c.1437C>T	p.Pro479Pro	rs1205194	N	N	N	N
** **	c.1499C>T	p.Ala500Val	rs41276418	Y (hetero) Score=25	Y (hetero) Score=21	Y (hetero)	Y (hetero)
** **	c.2223T>C	p.Pro741Pro	rs2747404	Y (homo) Score=25	Y (homo) Score=21	Y (homo)	Y (homo)
**LOC100287095**	**No Changes**	** **	** **	** **	** **	** **	** **
ZNF133 (NM_003434)	No Changes	** **	** **	** **	** **	** **	** **
**MGC44328 (NM_001004344)**	**c.89T>C**	**p.Leu30Pro**	rs1883938	**Y (homo) Score=27**	**Y (homo) Score=20**	**Y (homo)**	**Y(homo)**
C20orf12 (NM_018152)	c.40T>C	p.Leu14Leu	rs6035051	Y (hetero) Score=25	Y (homo) Score=25	Y (hetero)	Y (homo)
** **	c.801T>C	p.Cys267Cys	rs6035042	Y (homo) Score=29	Y (hetero) Score=13	Y (homo)	Y (hetero)
** **	c.1410G>C	p.Leu470Leu	rs6075337	Y (homo) Score=26	Y (homo)+ Score=21	Y (homo)	Y(hetero) +
**GCNT1P (NG_001039)**	**No Changes**	** **	** **	** **	** **	** **	** **
POLR3F (NM_006466)	c.840A>G	p.Thr280Thr	rs1055171	Y (homo) Score=26	Y (hetero) Score=13	Y (homo)	Y (hetero)
**RPL21P3 (NG_000976)**	**c.346delA**	**p.Arg116GlyfsX28**	**-**	N*	N*	**Y (hetero)**	**Y (hetero)**
RBBP9 (NM_006606)	No Changes	** **	** **	** **	** **	** **	** **
**RPS19P1 (NG_001295)**	**c.136C>G**	**p.Pro46Ala**	rs2009092	**Y (hetero) Score=12**	**Y (hetero) Score=5**	**Y (homo)**	**Y hetero)**
** **	**c.167G>A**	**p.Arg56Gln**	**-**	N*	N*	**Y (hetero)**	**N**
SEC23B (NM_032985)	c.490G>A	p.Val164Met	rs36023150	N	N	N	N
** **	c.1198T>C	p.Phe400Leu	-	N	Y (hetero) Score=8	N	Y(hetero)
** **	c.1276G>A	p.Val426Ile	-	N	N	N	N
** **	c.1467C>G	p.His489Gln	rs2273526	N	Y (hetero) Score=27	N	Y(hetero)
**LOC388789 (XM_939954)**	**c.48A>G**	**p.Arg16Arg**	rs12624935	N*	N*	**N**	**Y (hetero)**
DTD1 (NM_080820)	No Changes	** **	** **	** **	** **	** **	** **
**DUXAP7 (NG_004846)**	**c.447A>C**	**p.Gln149His**	rs6132094	N*	N*	**Y (hetero)**	**Y (hetero)**
** **	**c.664A>G**	**p.Thr222Ala**	rs6081271	N*	N*	**Y (homo)**	**Y (homo)**
** **	**c.665C>T**	**p.Thr222Ile**	rs6081270	N*	N*	**Y (hetero)**	**Y (hetero)**
** **	**c.716T>A**	**p.Ile239Lys**	**-**	N*	N*	**Y (hetero)**	**Y (hetero)**
** **	**c.719delA**	**p.Lys240LysfsX39**	**-**	N*	N*	**N**	**Y (hetero)**
** **	**c.720delA**	**p.Lys240LysfsX39**	**-**	N*	N*	**Y (hetero)**	**Y (hetero)**
** **	**c.721insA**	**p.Glu241ArgfsX25**	**-**	N*	N*	**Y (hetero)**	**Y (hetero)**
** **	**c.726_730dupGAAAA**	**p.Arg244ArgfsX37**	**-**	N*	N*	**Y (hetero)**	**Y (hetero)**
** **	**c.726_730dupGAAAA [****3****]**	**p.Arg244ArgfsX25**	**-**	N*	N*	**Y (hetero)**	**Y (hetero)**
** **	**c.943G>C**	**p.Val315Leu**	rs6132093	N*	N*	**Y (hetero)**	**Y (hetero)**
** **	**c.1124T>C**	**p.Val375Ala**	rs6081268	N*	N*	**Y (hetero)**	**Y (hetero)**
** **	**c.1156C>T**	**p.Pro386Ser**	rs6075382	N*	N*	**Y (hetero)**	**Y (hetero)**
**LOC100132358 (NC_000020)**	**c.274C>T**	**p.Gln92X**	rs6081369	**Y (hetero) Score=24**	**Y (hetero) Score=6**	**Y (hetero)**	**Y (hetero)**
**HSPC072 (AF161557)**	**No Changes**						
**LOC100270804**	**172C>T**	**p.Pro58Ser**	rs41309831	**Y (hetero) Score=13**	**N**	**Y (hetero)**	**N**
	**c.543G>T**	**p.Gly181Gly**	rs7262320	**Y (hetero) Score=26**	**N**	**Y (hetero)**	**N**
	**685_686insG**	**p. Glu229fxX23**	**-**	**Y (hetero) Score=14**	**N**	**Y (hetero)**	**N**
	**1343G>A**	**p.Arg448Arg**	rs80046336	**Y (hetero) Score=22**	**Y (hetero) Score=17**	**Y (hetero)**	**Y (hetero)**
	**1360_1364delAAGAA**	**p.Lys484fsX18**	**-**	**Y (hetero) Score=25**	**N**	**Y(hetero)**	**N**
**LOC100128496 (XM_001725705)**	**c.138C>G**	**p.Leu46Leu**	rs56743271	**Y (hetero) Score=19**	**N**	**Y (hetero)**	**N**
C20orf79 (NM_178483)	c.255C>T	p.Thr85Thr	rs1053834	Y (hetero) Score=25	N	Y (hetero)	N
	c.295C>T	p.Pro99Ser	rs1053839	Y (hetero) Score=20	N	Y (hetero)	N
SLC24A3 (NM_020689)	c.52_54delCGC*	p.Arg18del	-	N*	N*	Y (hetero)	Y (hetero)
	c.163G>A	p.Val55Ile	rs1569767	N	Y (hetero) Score=17	N	Y (hetero)
	c.369G>A	p.Ala123Ala	rs3790261	Y (Hetero) Score=26	Y (homo) Score=24	Y (hetero)	Y (homo)
	c.639G>A	p.Leu213Leu	rs3790278	Y (homo) Score=30	Y (homo) Score=15	Y (homo)	Y (homo)
	c.654T>C	p.Ile218Ile	rs3790279	Y (homo) Score=26	Y (homo) Score=23	Y (homo)	Y (homo)
**LOC100130264 (XM_001717962)**	**No Changes**						

Mutation score is provided for all NGS identified SNP. Bolded rows indicate predicted genes considered as non-coding regions by NextGENe Software (SoftGenetics). ^+^SNP identified by Sanger sequencing as heterozygous and by next-generation resequencing as homozygous. Manual reading of sequences generated by next-generation resequencing demonstrated 2 reads with a Guanine and 32 reads with Cytosine at base number 1,410 of *C20orf12*. *SNP located in a region that was not adequately covered (less than 5 reads) by NGS. ** Coverage was adequate to identify variant (between 5 and 20 reads), but was insufficient to produce a mutation call score of ≥5.

### Sequence capture and next generation sequencing of the PPCD1 common interval

Greater than 400,000 reads were obtained from each of the two DNA samples that were captured and sequenced, with an average read length of 350 bases. Approximately 80% of the reads mapped to the human genome, 35% of which mapped to the selectively enriched common PPCD1 interval, and about 20% of the reads did not match to the genome due to one or more factors, including the stringency of alignment settings. Alignment of the DNA sequence reads with the NCBI reference sequence resulted in at least 119 million matched bases from each sample. The average sequence coverage of the common PPCD1 interval was 28 fold for the affected individual and 19-fold for his unaffected son. Sequencing coverage of the 29 known and predicted genes (build 37.1) in the common PPCD1 interval varied significantly for the affected and unaffected individuals, ranging from zero to 70 fold.

### Sequence analysis using default parameters with Condensation Tool

Using the default parameters with Condensation Tool (Table 2, Default A), 430,175 matched reads were obtained in the affected individual’s DNA sample. A total of 67,599 DNA sequence variants were identified in the common PPCD1 support interval, of which 0.46% (311) were in the coding region of the 13 known genes. In the unaffected individual’s DNA sample, 370,400 matched reads were obtained, containing 27,448 DNA sequence variants in the common PPCD1 support interval, of which 0.56% (154) were coding region variants in the 13 known genes. NextGENe software was not able to distinguish intronic from exonic variants in the predicted genes as the entirety of the predicted genes was considered to be part of the non-coding sequence.

To determine the sensitivity of NGS, the sequence data generated by Sanger sequencing was compared to sequence data generated by NGS to determine what percentage of the exonic sequence variants identified in the 13 known genes and 16 predicted genes in the common PPCD1 interval with Sanger sequencing of the affected and unaffected individual’s DNA were identified with NGS. Eleven of the 17 (69%) exonic sequence variants previously identified by Sanger sequencing in known genes were identified in the affected individual with NGS. While two variants (c.52_54delCGC in *SLC24A3* and c.846T>C in *C20orf72*) were not reported due to insufficient coverage (less than 5 reads), four variants were not identified by NGS, even though they were located in regions of adequate coverage. Of the 28 exonic sequence variants identified in the 16 predicted genes, only 5 (17%) were identified by NGS. Nineteen of the remaining 23 variants were not identified due to insufficient coverage, while four were located in regions of adequate coverage but were not identified.

In the unaffected individual, NGS detected 14 of the 17 (82%) exonic variants identified by Sanger sequencing in the 13 known genes mapped to the common PPCD1 interval. Two of the three unidentified variants were located in regions of adequate coverage, while the other variant (c.52_54delCGC in *SLC24A3*) was not reported due to insufficient coverage. Of the 25 exonic sequence variants identified by Sanger sequencing in the 16 predicted genes, only two (8%) were identified by NGS. Nineteen of the remaining 23 variants were not identified due to insufficient coverage while four were located in regions of adequate coverage but were not identified.

### Sequence analysis using default parameters without Condensation Tool

Using the default parameters without the Condensation Tool (Table 2, Default B), 429,484 matched reads were obtained in the affected individual’s DNA sample. A total of 67,986 DNA sequence variants were identified in the common PPCD1 support interval, of which 0.53% (357) were coding region variants. In the unaffected individual’s DNA sample, 370,627 matched reads were obtained, in which 34,419 DNA sequence variants were identified in the common PPCD1 support interval, of which 0.54% (185) were coding region variants. In both the affected and the unaffected individual, 94% (16/17) of the exonic sequence variants identified by Sanger sequencing in the 13 known coding region genes mapped to the common PPCD1 interval were identified by NGS. In both individuals, the variant not identified by NGS was c.52_54delCGC in *SLC24A3*, which was located in a region of inadequate coverage. The other identified discrepancy between Sanger and NGS was the identification of the known SNP c.1410 G>C (rs6075337) in *C20orf12* in the heterozygous state by Sanger sequencing and in the homozygous state by NGS. Manual re-analysis of the reads generated by the NextGENe software revealed coverage of 34 reads at this base, with guanine present in 2 reads and cytosine present in the remaining 32 reads. Due to this 1:16 ratio, NextGENe software identified this variant as homozygous.

In the affected individual, 28 exonic sequence variants were identified by Sanger sequencing in the 16 predicted genes that map to the common PPCD1 interval, 9 (32%) of which were identified with NGS, while the remaining 19 were not identified due to insufficient coverage of the four genes in which the variants were identified (prothymosin, alpha pseudogene 3 [*PTMAP3*], ribosomal protein L21 pseudogene 3 [*RPL21P3*], ribosomal protein S19 pseudogene 1 [*RPS19P1*], and double homeobox A pseudogene 7 [*DUXAP7*]). In the unaffected individual, 25 exonic sequence variants were identified by Sanger sequencing in the 16 predicted genes, 6 (24%) of which were identified by NGS, with the remaining 19 sequence variants not identified due to insufficient coverage of the same four genes that were insufficiently covered in the affected individual.

Similar percentages of exonic sequence variants were identified in both known and predicted genes with NGS compared to Sanger sequencing in the affected (25/45; 56%) and unaffected (22/42; 52%) individuals. The 20 remaining sequence variants were located in the same five genes in each individual, with 19 of the 20 variants common to each individual.

### Sequence analysis using best adjusted parameters without Condensation Tool

By adjusting the default parameters without the Condensation Tool (Table 2, Best Adjusted), we were able to reduce the overall number of variants that were identified while still detecting each of the exonic sequence variants that had been identified by Sanger sequencing in the 29 genes in the common PPCD1 interval (provided there was adequate coverage of the gene region). The adjustments that were made were increasing the seed number (length of the sequence that is used to match to the reference genome), the move step (the number of bases between the seed start positions), the matching base percentage (the percentage of reads that need to match to the reference genome for alignment of the reads to the reference), and the mutation percentage (the minimum percentage of reads in which a sequence variant must appear before being consider a true variant). In the affected patient, 429,393 matched reads were obtained, in which 46,090 DNA sequence variants were identified in the common PPCD1 support interval. One hundred eighty-seven (0.40%) of the variants were located in the coding regions of the known and predicted genes, representing a 48% reduction in the number of coding region variants when compared to the number identified using the default parameters. Approximately one-half as many sequence variants were identified in the 360,870 matched reads in the unaffected individual (23,090) as were identified in the affected individual, representing a 33% reduction compared to the number identified using the default parameters, 0.54% (125) of which were coding region variants.

### Increasing specificity: Filter by Mutation Score

In an attempt to increase the stringency of the sequence variants identified by NGS, we reported a mutation call score for each sequence variant (Table 3). The mutation call score is a NextGENe software-generated score based on a mathematical algorithm that takes into consideration the level of coverage, the fraction of reads with the mutation, the probability of mismatch alignment, and potential false positive calls due to repeating sequences of bases known as homopolymer errors. Although SoftGenetics suggests a threshold mutation call score of 10, we felt that this was too stringent for our data set and therefore selected a threshold mutation call score of 5, which equates to an approximately 70% chance of an identified variant being real. Using the best adjusted parameters without the Condensation Tool, with a minimum overall mutation call score of 5, 9,492 DNA sequence variants were identified in the common PPCD1 support interval in the affected individual, of which 0.57% (54) variants were located in the coding regions of the 13 known genes. Each of the exonic coding region variants previously identified by NGS using the best adjusted parameters without the condensation tool were still identified after applying this additional screening criterion, with the exception of c.846T>C in *C20orf72* (inadequate coverage, 4 reads). Using the best adjusted parameters without the Condensation Tool, with a minimum mutation call score of 5, 5,627 DNA sequence variants were identified in the common PPCD1 support interval in the unaffected individual, of which 0.75% (42) variants were located in the coding regions of the 13 known genes. Two variants (c. 846T>C in *C20orf72* and c.843C>T in *PTMAP3*) that were identified by Sanger sequencing and were also previously identified by NGS were not identified after the application of a minimum mutation call score of 5.

## Discussion

The growing number of reports of the successful identification of pathogenic variants in human disease genes by targeted enrichment of a chromosomal locus followed by NGS are evidence of the utility of NGS as an efficient, cost-effective means to screen candidate genes [11,12,15]. While Sanger sequencing has been the preferred method over the past quarter-century for gene screening to identify a pathogenic sequence variant, it is an expensive and inefficient means to screen candidate loci that contain a moderate to large number of genes. Therefore, we were interested in performing NGS of a genetic region that had already been screened by Sanger sequencing, the common PPCD1 support interval, to determine whether NGS could in fact identify all of the exonic sequence variants that had been previously identified by Sanger sequencing.

Although NGS has quickly become the preferred technique among researchers for SNP identification, we identified several limitations of the technology. First, to identify all of the exonic variants in the common PPCD1 interval previously identified with Sanger sequencing, the filter parameters may need to be adjusted from their default settings. In the case of resequencing of the common PPCD1 interval, we knew what sequence variants were present in the selectively enriched chromosomal region a priori, and thus adjusted the filter parameters until each of the variants in regions of adequate coverage was identified. However, NGS will typically be used to screen regions using patient DNA samples that have not been previously sequenced, and thus investigators will not be able to optimize the filter parameters in the manner that we were.

Second, NGS was effective at identifying sequence variants only in regions in which the coverage was adequate. Factors that affect coverage, and therefore affect the likelihood of identifying a particular sequence variant, include the efficacy of selective enrichment of the region in which the variant is located, the sequencing efficiency of NGS (which depends on the platform that is used) and the reliability of sequence assembly and analysis [17]. A coverage simulation that was performed to determine the quality of data that is generated at various levels of coverage below the maximum achieved level demonstrated that coverage of at least 40× was necessary to achieve desirable SNP detection performance [9]. We found that coverage of at least fivefold was adequate to identify all of the variants previously identified with Sanger sequencing in the genes of the common PPCD1 support interval. However, the significantly greater number of sequence variants identified in the affected individual compared to the unaffected individual is likely attributable at least in part to the 15%–20% difference in the number of matched reads to the reference sequence between the two samples.

Third, NGS identified many more exonic sequence variants than were identified by Sanger sequencing, increasing the number of sequence variants that would need to be evaluated further by screening affected and unaffected individuals. To reduce the number of identified variants that represent false positives, we applied an overall mutation quality score, in this case the NextGENe software mutation score filter. Although the recommended threshold mutation call score is 10, corresponding to a 1 out of 10 chance of a variant being false, 6 of the 46 (13%) mutation call scores for the variants that we identified were under 10, prompting us to change the threshold to ≥5, which significantly reduced the number of identified variants. However, one factor to consider is the potential loss of real variants due to increased stringency: in our study, one variant identified by Sanger sequencing in the affected patient and two variants identified with Sanger sequencing in the unaffected patient that NGS identified using the best adjusted parameters without the condensation tool were not identified when the mutation score filter was applied. As the mutation call score is dependent to large degree on the level of coverage, the failure to identify these variants due to insufficient coverage highlights the fact that this filter be used only in regions of sufficient coverage.

In conclusion, we report that NGS is able to reproduce sequencing data generated through Sanger sequencing in a significantly shorter period of time, and with far less expense. As the NGS platforms continue to evolve, we expect that many of the limitations of the technology that researchers face currently will be resolved though improved sequence capture and sequencing methods, as well as more sophisticated techniques of sequence assembly and analysis.
